# “How do I keep this live in my mind?” Allied Health Professionals’ perspectives of barriers and enablers to implementing good clinical practice principles in research: a qualitative exploration

**DOI:** 10.1186/s12913-023-09238-5

**Published:** 2023-03-30

**Authors:** Rachel Wenke, Shelley Roberts, Rebecca Angus, Maame Amma Owusu, Kelly Weir

**Affiliations:** 1 School of Health Sciences and Social Work, Griffith University Gold Coast, Gold Coast, Australia; 2 Allied Health and Rehabilitation Services, Gold Coast Health Southport, Southport, Australia; 3 Menzies Health Institute Queensland, Griffith University, Gold Coast, QLD Australia; 4 Office of Research Governance and Development, Gold Coast Health Southport, Southport, Australia

**Keywords:** Research conduct, Allied health, Good clinical practice, TDF

## Abstract

**Background:**

Allied health professionals (AHPs) engaged in research are expected to comply with Good Clinical Practice (GCP) principles to protect participant safety and wellbeing and enhance data integrity. Currently, few studies have explored health professionals’ perceptions of implementing and adhering to GCP principles in research with none of these including AHPs. Such knowledge is vital to guide future interventions to increase adherence to GCP principles. This study aimed to identify the barriers and enablers AHPs experience when applying GCP principles to research conduct in a public hospital and health service, as well as their perceived support needs.

**Methods:**

The study used a qualitative descriptive study approach guided by behaviour change theory. AHPs currently undertaking ethically approved research within a public health service in Queensland, Australia were interviewed to explore barriers and enablers to adherence to GCP principles and support needs, with interview questions guided by the Theoretical Domains Framework (TDF). The TDF was chosen as it allows for a systematic understanding of factors influencing implementation of a specific behaviour (i.e., GCP implementation) and can be used to inform tailored interventions.

**Results:**

Ten AHPs across six professions were interviewed. Participants identified both enablers and barriers to implementing GCP across nine domains of the TDF and enablers across three additional domains. Examples of enablers included strong beliefs about the importance of GCP in increasing research rigour and participant safety (i.e. from TDF - beliefs about consequences); applying clinical skills and personal attributes when implementing GCP (i.e., skills), available training and support (i.e., environmental context and resources); and alignment with their moral sense to ‘do the right thing’ (i.e., professional identity). Barriers to GCP implementation were generally less commonly reported but included reduced time to implement GCP and a sense of ‘red tape’ (i.e., environmental context and resources), a lack of knowledge of GCP principles (i.e., knowledge) and a fear of making mistakes (i.e., emotions), and varying relevance to individual projects (i.e., knowledge). Suggestions for support were identified beyond training, such as physical resources (e.g., prescriptive checklists, templates and scripts), additional time, and regular one-on-one mentoring support.

**Conclusion:**

Findings suggest that while clinicians recognise the importance of GCP and want to implement it, they report barriers to its practical implementation. GCP training alone is unlikely to address these barriers to implementing GCP in daily practice. Findings suggest that GCP training may be more useful to AHPs when it is tailored to the allied heath context and supplemented with additional supports including check-ups from experienced researchers and access to prescriptive resources. Future research however is needed to investigate the effectiveness of such strategies.

**Supplementary Information:**

The online version contains supplementary material available at 10.1186/s12913-023-09238-5.

## Background

Conducting research in a way that protects participants and yields credible and reliable results is central to Good Clinical Practice (GCP). The GCP guidelines are an international ethical and scientific quality standard for designing, conducting, recording, and reporting trials that involve the participation of human subjects produced by the International Conference on Harmonisation [1]. While the name “Good Clinical Practice” suggests these guidelines relate to “clinical practice”, in contrast, they describe a set of principles for how to conduct good clinical “research” in everyday practice. The GCP guidelines were originally designed for drug or investigational product clinical trials, however they are now widely applied to other intervention types [2], including behavioural interventions. This means any research design that prospectively assigns human participants to an intervention including observational cohort studies or qualitative designs are recommended to comply with GCP principles [3]. Due to this broad definition of what constitutes a clinical trial, an increasing number of investigators involved in healthcare research are required to ensure their research adheres to GCP principles including allied health professionals (AHPs). AHPs, who include physiotherapists, occupational therapists, psychologists, speech pathologists, audiologists, social workers, dietitians and podiatrists, among others [4], are being increasingly encouraged to participate in and lead research [5, 6] and subsequently adhere to these GCP guidelines in research conduct.

Despite this, there has been no research to our knowledge exploring their perspectives or experiences in doing so. While we found three studies which have investigated health professionals’ perceptions and experiences of adhering to GCP standards, none of these studies included AHPs.

The only research to date exploring perceptions of health professionals regarding GCP guidelines has been involving medical professionals. Swezey et al. [7] interviewed 10 medical investigators with at least 5 years’ experience working in phase 3 clinical trials of drugs, biologics and/or medical devices and 13 research sponsors to gain their perceptions of good clinical conduct and training needs in GCP principles. This study highlighted that GCP training should be presented in a way that is engaging to participants and focuses on daily application in practice [7]. Interviewees from a follow up study in the same context suggested that completing GCP training alone was insufficient to prepare the conduct of a clinical trial and a more tailored rather than repetitive “one-size fits all” approach to GCP training may be required to meet the needs of investigators [8]. This may include access to mentorship and opportunities to receive in real-world training [8]. In a descriptive editorial piece, Cronin et al. similarly described a more tailored approach used in a US paediatric medical setting to improve understanding and application of GCP into everyday work for their investigator group [9]. This approach included the development of an institutional GCP training module which focused on operationalising GCP principles around each stage of a study and highlighting relevant procedures and resources for each of these stages within the paediatric clinical research [9].

While limited studies from medical teams are emerging, there is a need to explore the barriers and enablers to adhering to GCP principles and the support needs more systematically across other health professionals, such as AHPs, within different healthcare contexts and geographical locations beyond the USA. Such knowledge will help to further understand factors influencing health professionals adherance to GCP to subsequently guide tailored interventions that promote adherance to GCP guidelines in everyday research practice. While a body of research currently exists exploring the general barriers and enablers AHPs have regarding research engagement more broadly [10, 11–14] including the impact of time, skills and resources; to our knowledge no studies exist exploring the specific barriers and enablers to AHPs implementing GCP in research.

Further research inquiry must also recognise that successful implementation of GCP principlesrequires health professionals to change their behaviour in how they conduct and manage their research in everyday practice. Applying behaviour change theory may therefore be of value when developing tailored approaches to enhance GCP adherence in the clinical setting and guide the systematic evaluation of health professionals’ needs. One useful framework to understand barriers and enablers to implementing a specific behaviour is the Theoretical Domains Framework (TDF) which is an integrative framework of over 30 behaviour change theories [15]. The TDF explores the influence of 14 domains on behaviour change which include: knowledge; skills; social/professional role/identity; belief about capability; optimism; belief about consequences; reinforcement; intentions; goals; memory, attention and decision processes; environmental context and resources; social influence; emotion; and behavioural regulation (defined in Supplementary File 1). Another widely used model of behaviour change complementary to the TDF is the COM-B system [16]. This model includes three factors; capability, motivation and opportunity are necessary to change health professional’s behaviour [16] and can be used to interpret findings from the TDF according to these three factors to help develop tailored interventions which promote behaviour change [14, 17].

Our team has previously shown the value of using both the Theoretical Domains Framework (TDF) and COM-B system of behaviour change to explore barriers and enablers to general research participation among AHPs and developing intervention strategies [14]. Interviews of 21 AHPs in a public health service identified barriers and enablers to research participation within nine domains of the [14]. Most enablers were related to the motivation or opportunity constructs of the COM-B including positive beliefs about the consequences of participating in research while predominant barriers related to environmental context and resources, perceived reduced capability and emotional responses including the sense of being [14]. Despite the potential usefulness of behaviour change theory demonstrated by Wenke et al. [14], there has been no systematic evaluation using such frameworks to examine the barriers and enablers specific to implementing GCP guidelines by health professionals. Such research is vital to inform future interventions to support implementation of GCP principles to ensure that research is conducted according to international standards to yield quality data and ensure the wellbeing and privacy of participants is upheld.

## Aim

This study aimed to identify the barriers and enablers AHPs face in implementing GCP principles when conducting research in a public health service, and their perceived support needs.

## Methods

This study used a qualitative descriptive design [18] guided by behaviour change theory [15, 19] and is reported according to the reporting standards for qualitative research (COREQ) [20]. It was approved by the Gold Coast Hospital and Health Service (GCHHS) Human Research Ethics Committee (LNR/2021/QGC/73,671), with all methods performed in accordance with the relevant guidelines and regulations including the Declaration of Helsinki.

### Study setting

The study took place within the Gold Coast Hospital and Health Service (GCHHS), a public, regional tertiary health service in Queensland, Australia that employs approximately 1,200 allied health staff. Three part time Research Fellows currently work within the health service, mentoring AHPs throughout the research cycle. The health service expects all clinician researchers to undertake GCP training and provides free GCP training workshops (Sophie Mepham GCP™, TransCelerate accredited to ICH GCP E6 R2).

### Participants

AHPs working at GCHHS were invited to participate if they: (1) worked within audiology, dietetics, music therapy, occupational therapy, physiotherapy, podiatry, psychology, social work, or speech pathology; and (2) were listed as a principal or lead investigator on an ethically approved research project (or a project in preparation) at GCHHS in the past three years (2018 onwards). Clinicians who met the above criteria were identified through internal information sources on current ethically approved research (i.e., research metric registers). AHPs were emailed an invitation to participate and a Participant Information and Consent form by the Principal Investigator. Participants were recruited using convenience sampling to firstly identify clinicians who met the criteria, and purposive sampling to ensure a diverse sample was selected (i.e., varying in profession and research experience). Informed written consent was obtained from all participants.

### Study procedure

Semi-structured interviews were conducted to explore the barriers, enablers, and support needs of AHPs in implementing GCP principles in their research. The interviews were audio-recorded and conducted either in person in a quiet office in the hospital or via videoconference (i.e., Microsoft TEAMs). Interview questions were developed by RW with consultation from the research team and guided by the 14 domains of the TDF and a previous related [14]. Additional questions regarding support needs were also included to address the research aim (Supplementary File 2: Interview guide).

Interviews were conducted by the lead investigator RW, except for one where there was a mentoring relationship between RW and the clinician (subsequently conducted by RA). In all other occasions, RW had a working professional relationship with the interviewees but was not actively mentoring them on any research projects. Both interviewers were experienced researchers with an AHP background and were employed by the same health service as the interview participants.

Demographic data collected included years of clinical experience, gender, professional background, and previous research experience including any post-graduate research qualifications.

### Data analysis

All interview data were transcribed using a professional transcription service and analysed using NVIVO software (version 12). Deductive analysis was used to categorize barriers and enablers identified in the interview data into one or more of the 14 TDF domains, combined with a general inductive approach to identify emergent subcategories by grouping related quotes within each domain [21]. Support needs were coded using inductive coding and content analyses. The analysis was led by RW, with all codes reviewed by a second project team member (KW, SR, or RA). Any discrepancies were discussed until a consensus was reached. Following these analyses, the most frequently reported barriers and enablers, as determined by the number of sources and references coded to that specific barrier or enabler were further synthesized by RW mapping them onto the three constructs of the COM-B system: capability, opportunity, or motivation [16]. The COM-B system, a model of behaviour change, cites these three factors are necessary to change behaviour [16], thereby providing a useful theoretical framework to guide future intervention development to change health professionals’ behaviour in relation to GCP implementation. While a convenience sample was used, data saturation was achieved with no new categories being found when analysing the final two participants.

## Results

### Participants

A total of 24 AHPs were identified as meeting inclusion criteria. Thirteen AHPs were invited to participate via email, with 10 consenting. Three clinicians did not respond due to being on leave or having left the health service. Interviews took on average 32 min (range 18–42 min) with eight of these interviews completed face to face and two via videoconference. Participant demographics are reported in Table 1.

**Table 1:** Participant Demographics

Variable	Number (or mean as indicated)
Gender	
Female	9
Male	1
**Years of clinical experience** (mean)	12.6 (range 0.5–20)*
**No. of projects led** (mean)	3.3 (range 1–10)
**Current Clinical Level**:	
Entry level clinician	2
Senior clinician	5
Team leader	3
**Research qualifications**	
Enrolled in PhD	4
Completed PhD	1
Research related Master’s Degree	2
Enrolled in Master of Philosophy	1
Nil formal qualifications	2
**Profession**	
Dietetics	2
Occupational Therapy	1
Physiotherapy	2
Psychology	1
Social Work	2
Speech Pathology	2

*Note one of the participants with 0.5years clinical experience had moved to a non-clinical research role and working in this role at the time of the interview

## Barriers and enablers to implementing GCP

Participants generally reported more enablers than barriers towards implementing GCP within their research. Participants cited barriers and/or enablers across all 14 TDF domains (see supplementary file 3). Most comments that included both barriers and enablers related to eight of the domains, whie four additional domains. Table 2 further describes these be described under the categories capability, opportunity, and motivation; of the COM-B system. A summary of these factors mapped according to the COM-B can be found in Table 2. For further details regarding reported barriers and enablers across all 14 domains including frequency of mention and number of sources, please refer to Supplementary File 3.

**Table 2:** Factors which influence allied health implementation of GCP

Capability	Opportunity	Motivation
**Knowledge** • Knowledge of research conduct • Relevance of training **Memory, attention and decision processes;** • Keeping things fresh in mind	**Environmental Context and Resources** • Time and funding available to undertake training and implement principles • Local infrastructure and support **Social influence** • Support from peers and more experienced researchers	**Beliefs about consequences, Goals, Optimism** • Perceived benefits and desire towards implementing GCP **Emotions** • Emotional reactions of fear/anxiety to making mistakes **Professional identity, reinforcement** • Moral sense to do the right thing and avoid others’ mistakes **Beliefs about capabilities** • Confidence level to implement

## Capability

### Knowledge

Several participants reported increased knowledge about GCP from participating in GCP training: *“It was a four-hour workshop that we had to do, which was fantastic in just introducing me to all of these concepts that I didn’t even know about”* (P10). Fewer reported gaining this knowledge from university courses or completing a higher research degree: *“I guess it’s just been drummed through my graduate training…” (P03).*

Most participants reported sound knowledge of what constitutes good research conduct in a general sense: *“Good conduct in research obviously means conducting research that’s been ethically approved and complying with how you got it approved with the protocol, making sure you’re considering patient safety, confidentiality, all of that” (P09).* However, a number of participants were unfamiliar with the term GCP, despite reporting adherence to its concepts: “*I would say that the senior researchers that I work with don’t use the same language about Good Clinical Practice and this definition…I don’t think that they label it that way but they’re concepts they talk about all the time”* (P01), while others were unclear what the principles of GCP were: “*I wouldn’t say that I’m actually overly familiar if there are any certain guidelines or principles.”* (P04).

Another knowledge-related barrier to using GCP was a lack of awareness, coined by participants as *“you don’t know what you don’t know”* (P02), which impacted their ability to source information: “*I don’t know what I don’t know, so being able to find information to make it easier to adhere to these [GCP principles]” (P07).*

### Skills

Clinicians reported personal attributes that were helpful in implementing GCP: *“I’m quite detail-orientated, a little bit perfectionistic, so that probably helps me to ensure that these things are met each time I design a research project.” (P09).* Clinicians also said their current clinical skills transferred into useful skills when adhering to GCP: *“As a hospital clinician, we already are thinking about confidentiality, and assuring that our work is confidential and it’s stored and managed correctly. So I bring that skill set to this - which is transferable to these principles” (P02).* Clinicians however reported barriers in specific skills of implementing GCP principles, for example, “*how to recruit ethically because I found that tricky. I feel it was a bit of a sales pitch and I’m not like that at all in my personality” (P08)*.

### Memory, attention and decision processes

In this domain having specific processes set up ready to follow were found to be helpful including detailed instructions and other prescriptive guides, templates and scripts; as one participant commented, *“So, having a script, having practiced that. Having everything set up ready to go… It got better over time in terms of adhering to the principles.”* (P08). The use of reminders or other strategies to bring GCP principles to the forefront of the busy clinician’s mind were also reported: *“when life gets busy and the years go on, probably just having some sort of process that just jogs your memory along the way would be good*” (P03). Some clinicians said it became more automatic to implement GCP over time as they became used to applying the principles: *“I think when you start out in research, and it’s like a whole different world of things that you just try and navigate and possibly not doing things correctly all the time, but once you know what you need to do it just makes it easier. So being able to stick to GCP principles just becomes second nature I guess.” (P07).*

Barriers to implementing GCP in this domain included difficulty remembering what was learnt in GCP training, “I mean I felt like I did the training once and thought, oh yeah that was good and then haven’t really thought about it since …. I can probably only remember the parts of the training that fitted to my assumption in the first place” (P01) and keeping the principles live at the forefront of your mind: *“When things are really busy, and you’re rushing between things, I still haven’t figured out ‘how do I keep this live in my mind?’ (P02).*

## Opportunity

### Environmental context and resources

Most comments under this domain described barriers related to inadequate time or funding to attend training or implement the principles in their projects. For example one clinician reported, “*Obviously it’s hard for everyone to do it [training] given the time commitment… in reality when they’ve got full caseloads, it’s difficult to get a number of people offline to do it.”(*P07), while another described, “*funding or the resources to be able to do it and probably not allowing enough time and resources to actually do some of those things”* (P04) Another clinician described, *“The biggest barrier to me is time. So if I don’t have time to be able to do these things, I’m probably not going to do it, or I’m going to rush it, and possibly something would get missed”* (P03).

Other barriers related to the accessibility of resources including not knowing where current resources or templates are located: “*coming in as a clinician…there’s nowhere to go to find this information. I know there’s that website that’s meant to have lots of things on it like templates and forms but it’s not super easy to find things*” (P08); as well as comments regarding current training not being relevant to their project design: *“I found a lot of it seemed very specific to clinical trials, which was I think a lot less relevant to the work in particular that I do*” (P05).

Existing research infrastructure within the health service including training and resources from the Research Office were described as enablers to GCP implementation, *“especially now that the Research Office has got people employed …who are giving us GCP training and support and resources. I think that’s really important.* (P09).

### Social influences

Several clinicians noted the benefit of support from more experienced researchers including local Research Fellows and PhD supervisors to implement GCP: *“I think having support people and people that are experienced in research does make it a lot easier (P09).* Support from peers or colleagues also enabled GCP implementation: “*In terms of support…we have a good research peer environment here at Gold Coast, so there are people who are there to ask questions and to learn from experience” (P06)* and *“having teams share responsibility and have regular communication”* (P02). A smaller number of clinicians reported that there was a lack of support from their professional teams due to limited research experience: *“I think the fact that our… department is really evolving in their interest in research, it’s not, from my perspective, well established; it’s quite new, and foreign… so not a lot of local support, but I know it’s not far away”* (P10).

## Motivation

### Beliefs about consequences

Almost half of AHPs felt implementing GCP principles could restrict the logistics of some projects: *“There is risk that good research might not be started in the fear of… too much red tape or too many hoops to jump over”* (P04), while some felt there was a risk of implementing the principles in a superficial way: “*I just think generally, when we have standards and principles, it’s possible to tick boxes without really engaging with what we’re doing.*” (P02).

Almost all clinicians reported two enabling beliefs about the consequences of implementing GCP, being improved rigour, replicability, and ethical conduct of the research: *“It means that you get good quality research as well, like good rigour”* (P03), and improved participant safety: *“Hopefully it means that the studies are safer, that the participants’ privacy or confidentiality is protected, that there’s fewer adverse events, that there’s just fewer issues in general”* (P09). Some AHPs said implementing GCP was important to avoid other negative consequences: *“There’s serious consequences if you don’t, as well as it doesn’t make you a reputable researcher if you’re dodgy [laughs]. So yeah, it has consequences for the organisation as well and for yourself and obviously for participants, so yeah, it’s kind of important that you stick to it”* (P07).

### Emotions

While some perceived consequences encouraged GCP implementation, clinicians commonly reported anxiety or fear of negative consequences as a result of making a mistake: “*It does make you a little bit anxious. There’s lots of things that maybe you haven’t considered and the consequences for not adhering to it”* (P07). Clinicians also reported feeling pressured or overwhelmed amidst their other priorities: “*because there is a lot to consider, it can be overwhelming because you’re thinking ‘oh gosh, when am I going to get time within the constraints of what I’m already doing and the research?’”* (P04).

Clinicians reported a sense of comfort or reassurance that GCP principles helped guide their research conduct: *“I think it’s reassuring to know that these structures are in place… that we as researchers should be adhering to these principles”* (P02), as well as a sense of responsibility: *“I certainly feel a sense of responsibility and a sense of duty to ensure that I’m conducting research in a way that’s ethical and safe* (P02).

### Beliefs about capabilities

While most clinicians felt confident about implementing GCP principles, with one commenting “*I don’t think there’s anything that’s too difficult to adhere to”* (P07), several clinicians identified certain aspects of implementing GCP where they perceived they needed more development: *“My protocol writing skills could be developed. Honestly, I could refine all of my research skills and there’s probably not an area that doesn’t need development if I’m 100 per cent honest*” (P02). Training was seen to help confidence: “*I just found it personally really valuable to attend the training and I’d say that helped increase my confidence in my research*” (P08).

### Social professional role and identity

Other enablers to implementing GCP included it being part of clinicians’ responsibility as a Principal Investigator: *“As an investigator I think everybody has a responsibility to adhere to…good clinical practice” (P06)*, and that it aligned with their values to do the right thing: “*I like to have good principles in my everyday life, I don’t like to…be unethical about things, so I’m not usually cutting corners* (P07).

### Optimism, goals and reinforcement

Participants also reported enablers to implementing GCP related to Optimism, Goals and Reinforcement. For example, participants expressed a general optimism towards being able to implement GCP *“I know personally, I will be able to adhere to every step of it.”* (P10). Within the domain of Goals, most participants reported strongly wanting to adhere to GCP and for their research to be credible; *“ I feel very strongly that I want to adhere to those principles, and I feel very strongly that I want my research to reflect … my perception of the values that underpin these standards*” (P02). Lastly, some participants also reported as a reinforcing enabler to implementing GCP - learning from examples of poorly conducted research so that they would not repeat such mistakes, *“I have witnessed and seen research that hasn’t been conducted in a good clinical practice – not here…. So that’s made me a little bit concerned that I want to make sure that I don’t cut any corners” (P06).*

## Support needs

Seven categories emerged from the interviews regarding future suggestions of support needs to implement GCP. These are outlined in Table 3.

**Table 3:** Summary of support needs

Support need categories	Number of Sources	Total number of mentions
1. Physical or Financial Resources		
Checklist, flowchart or manual specific to research designs	9	15
List of resources you can go to	3	3
Examples of templates, applications, folders & protocols	3	5
Directory of who to go for support including GCP contact	3	3
Extra time to complete training and implement	3	4
Protocol with extra information about principles and processes	2	4
Reminders when to do things (e.g., annual reports)	1	2
2. One on One support		
Someone to check up on project in non-threatening way (i.e., ‘supportive’ auditing)	8	18
Research fellows or senior researcher to provide guidance/advice on how to implement principles	3	4
3. Increase awareness and visibility of GCP	6	11
4. Hearing other people’s experiences	1	2
5. GCP Training recommendations		
Training should be early	3	5
Tailor to research design or allied health	5	10
Need support how to implement what’s learnt from training	5	6
Being aware of training	3	5
Annual training refreshers	4	5
6. Format of resources		
Having online resources, ‘one stop shop’	8	11
Videos	6	7
Simple and easy to understand, not too long	4	9
Relate it to being a clinician	2	3
Needs to be accessible	2	3
Easier if can read it	3	3
7. Suggestions to make GCP interventions sustainable		
Make sure HHS values it (GCP) & invests in resources	3	7
Embed into critical part of planning and conducting research	3	3
Accessible & regularly updated	3	5

## Discussion

The present study was the first to explore AHPs’ perceived barriers and enablers to implementing GCP standards in research projects as well as their support needs regarding GCP implementation. This research highlights the usefulness of using behaviour change theory including the TDF and COM-B to understand the factors which influence health professionals in implementing GCP. Findings revealed clinicians reported barriers and enablers to implementing GCP principles across each of the TDF domains suggesting that factors beyond capability including opportunity and motivation can influence day to day implementation.

Historically, GCP training has been the main stay intervention to support clinician’s implementation of GCP. While training is identified as a useful strategy to enhance capability, in particular knowledge and skills [22], it may not address all barriers to GCP implementation identified in the present study. For example typical GCP training does not address barriers related to the TDF domains of memory, attention and decision making; environmental context and resources; or emotions (i.e., fear of making mistakes). As such, our research highlights supplementary strategies targeting these specific barriers are required. Examples of potential strategies to address barriers across the COM-B are found in Table 2. Previous research has identified similar enhancements, including incorporating more tailored practical discussion in GCP training and mentorship for new investigators [7], however such tailored strategies to support GCP implementation have had limited investigation [9]. With an expanding range of investigators with varying levels of experience and support now expected to apply GCP [2], attention to strategies that address the unique enablers and barriers to GCP implementation for them individually and within their team must be considered. A “one size fits all” approach to support is unlikely to be effective [8, 9].

**Table 4:** Example of theory-informed strategies to support behaviour change in implementing GCP

COM-B component	TDF domain	Barrier	Potential intervention to support behaviour changes needed for GCP implementation
Capability	Knowledge	Unfamiliarity or lack of awareness of concept of GCP& how to access information	Increase visibility and awareness about GCP through linking with existing processes (e.g., receipt of ethics approval clinicians receive information about GCP)
			Create resources which are easily accessible to clinicians about GCP and relevant processes
	Skills	Reduced skills in specific areas including recruitment	Link in with more experienced researcher to check-in on projects’ GCP adherence. Have specific scripts and examples of GCP processes available online for clinicians to refer to.
	Memory, Attention, Decision Processes	Difficulty remembering what was learnt in GCP training	Have online resources that can be accessed after training and referred to including checklists of how to apply GCP principles specific to different research designs across the research cycle.
Opportunity	Environmental Context and Resources	Training not relevant to certain project designs	Include non-clinical trial designs (as relevant to Allied Health) in GCP training
	Social Influences	Lack of support from professionals’ teams	Link in with more experienced researchers and peers to support GCP adherence
Motivation	Emotions	Anxiety or fear over making a mistake	Link in with more experienced researchers and peers to support GCP adherence and offer guidance
		Sense of overwhelm to implement amidst other priorities	Clinicians given dedicated time prior to commencing research project to ensure project is “GCP-ready”. Access to templates and examples to reduce time in preparing projects to adhere to GCP standards.
	Beliefs about capabilities	Reduced confidence in implementing certain aspects of GCP	Link in with more experienced researcher and refer to online resources, videos and examples, templates to support implementation.

The present study also offers unique insights into AHPs’ perspectives of GCP in a range of research designs. AHP participants felt GCP was important; a finding that echoes previous research involving medical practitioners [7]. However, while AHPs generally had good knowledge about research conduct, drawing from some of their clinical knowledge, many reported a lack of confidence in their ability to apply their knowledge to specific aspects of their projects. A key enabler to implementation of GCP principles which may potentially address this lack of confidence, was the support of social influences including peers, colleagues, and more experienced researchers. This power of social influences, particularly peers, is consistent with an earlier study of AHPs’ research engagement [14].

## Limitations and future directions

Participants recruited in this study had varied research experience and professions, however came from a single health service. While six prevalent allied health professions were represented in the study’s sample, only 10 participants were involved and therefore some professions including pharmacy and others with a smaller proportion of staff in the hospital (i.e., podiatry and music therapy) were not represented. As such, future research should explore perspectives from other professional and geographical contexts to determine whether similar themes arise. Evaluating the feasibility and effectiveness of some of the identified strategies (e.g., as listed in Table 4) which aim to enhance adherence to GCP would be useful. This evaluation is currently underway by the research team.

## Practical implications

The present study suggests GCP training alone is likely insufficient for clinicians to implement GCP principles in their research and assistance may need to be tailored to the research design utilised, context and experience of the researcher. For example, extending training beyond clinical trials to other research designs, including observational research, would be useful. Additional support and mentoring in GCP may be required across different research phases, particularly for novice researchers. This may include embedding periodic GCP audits or ‘check-ins’ by a more experienced researcher, such as a Research Fellow, in a non-threatening manner to minimize reported anxiety, particularly if it is seen as usual practice and a learning opportunity. This strategy may also identify any potential areas of non-adherence early before they become a breach in ethical conduct. Access to more prescriptive resources to support implementation (e.g., checklists, templates, scripts) may further address barriers related to confidence and fear of making mistakes whilst creating efficiencies; thus, potentially addressing barriers relating to reduced time and resources. A whole systems approach may be beneficial, whereby health services identify what other mechanisms are available to keep GCP principles at the forefront of researchers’ minds, and to ensure it is not simply considered a tick box exercise of attending the training and forgetting to implement daily. This may be in the form of reminders, further integration with existing systems, and embedding GCP guidelines into learning earlier when supported by research fellows or mentors.

## Conclusion

This study has shown that while AHPs recognise the importance of GCP principles and express a desire to implement it, they report barriers in its practical implementation. These challenges may be addressed by supplementing the level of support in GCP implementation beyond standard training. Further clarification of these strategies and evaluation of their effectiveness should be investigated to support health professionals implementing GCP principles in research, to ultimately protect the safety and wellbeing of participants involved in research and the integrity of data being collected.

## Electronic supplementary material

Below is the link to the electronic supplementary material.

Supplementary file 1: Definitions of the TDF domains taken from Atkins et al., and Cane et al (Atkins et al., 2017; Cane, O?Connor, & Michie, 2012)

Supplementary File 2: Semi-structured Interview Guide

Supplementary Material 3

## Data Availability

The datasets used and/or analysed during the current study are available from the corresponding author on reasonable request.

## List of abbreviations

GCP: Good Clinical practice
AHP: Allied Health Professionals
