# Supplementary material for: “How do I keep this live in my mind?” Allied Health Professionals’ perspectives of barriers and enablers to implementing good clinical practice principles in research: a qualitative exploration
Source: BMC Health Serv Res. 2023 Mar 30;23:309. doi: 10.1186/s12913-023-09238-5 (PMC10064695; doi:10.1186/s12913-023-09238-5)
Supplement: Supplementary file 1 — Definitions of the TDF domains taken from Atkins et al., and Cane et al (Atkins et al., 2017; Cane, O?Connor, & Michie, 2012) [file 12913_2023_9238_MOESM1_ESM.docx]

Supplementary file: Definitions of the TDF domains taken from Atkins et al., and Cane et al (Atkins et al., 2017; Cane, O’Connor, & Michie, 2012)

| **Domain** | **Definition** |
| --- | --- |
| **1. Knowledge** | An awareness of the existence of something |
| **2. Skills** | An ability or proficiency acquired through practice |
| **3. Social/Professional Role and Identity** | A coherent set of behaviours and displayed personal qualities of an individual in a social or work setting |
| **4. Beliefs about Capabilities** | Acceptance of the truth, reality, or validity about an ability, talent, or facility that a person can put to constructive use |
| **5. Optimism** | The confidence that things will happen for the best or that desired goals will be attained |
| **6. Beliefs about Consequences** | Acceptance of the truth, reality, or validity about outcomes of a behaviour in a given situation |
| **7. Reinforcement** | Increasing the probability of a response by arranging a dependent relationship, or contingency, between the response and a given stimulus |
| **8. Intentions** | A conscious decision to perform a behaviour or a resolve to act in a certain way |
| **9. Goals** | Mental representations of outcomes or end states that an individual wants to achieve |
| **10. Memory, Attention and Decision Processes** | The ability to retain information, focus selectively on aspects of the environment and choose between two or more alternatives |
| **11. Environmental Context and Resources** | Any circumstance of a person's situation or environment that discourages or encourages the development of skills and abilities, independence, social competence, and adaptive behaviour) |
| **12. Social influences** | Those interpersonal processes that can cause individuals to change their thoughts, feelings, or behaviours |
| **13. Emotion** | A complex reaction pattern, involving experiential, behavioural, and physiological elements, by which the individual attempts to deal with a personally significant matter or event |
| **14. Behavioural Regulation** | Anything aimed at managing or changing objectively observed or measured actions |

All definitions are based on definitions from the American Psychological Associations’ Dictionary of Psychology (23)

Atkins, L., Francis, J., Islam, R., O’Connor, D., Patey, A., Ivers, N., . . . Michie, S. (2017). A guide to using the Theoretical Domains Framework of behaviour change to investigate implementation problems. *Implementation Science, 12*(1), 77. doi:10.1186/s13012-017-0605-9

Cane, J., O’Connor, D., & Michie, S. (2012). Validation of the theoretical domains framework for use in behaviour change and implementation research. *Implementation Science, 7*(1), 37. doi:10.1186/1748-5908-7-37
