# Supplementary material for: “How do I keep this live in my mind?” Allied Health Professionals’ perspectives of barriers and enablers to implementing good clinical practice principles in research: a qualitative exploration
Source: BMC Health Serv Res. 2023 Mar 30;23:309. doi: 10.1186/s12913-023-09238-5 (PMC10064695; doi:10.1186/s12913-023-09238-5)
Supplement: Supplementary file 2 — Semi-structured Interview Guide [file 12913_2023_9238_MOESM2_ESM.docx]

## Supplementary File 2:

**Semi-structured Interview Guide**

The following are example questions to be asked during the interview.

| **Domain** | **Example/Guiding Questions (based on TDF)** |
| --- | --- |
| **General introductory** | What does good conduct in research mean to you? |
| **Knowledge** | How familiar are you with the concept of GCP (Good Clinical Practice) in research?  What are some of the principles of GCP?  *Interviewer will provide a working definition of GCP and list of abbreviated GCP principles to set the rest of interview in context.* |
| **Motivation and goals** | How much do you want to or/feel you need to adhere to principles of GCP when conducting your research? |
| **Social influences** | Do you have any support or encouragement from others to adhere to principles of GCP?  (prompt- peers, managers, other professional groups, patients, research office) |
| **Emotion** | How does it make you feel when you think about adhering to principles of GCP when conducting research? |
| **Social/professional role & identity** | Do you think it is expected of you as part of your role as an investigator that you adhere to GCP principles when doing research? Why/Why not.  Does this influence what you think about engaging in research? |
| **Behavioural regulation** | What actions (if any) would you need to take to adhere to principles of GCP when you conduct your research project?  Are there any things you can think of that would encourage achieving that? |
| **Beliefs about consequences** | What do you think are the outcomes to adhering to the principles of GCP in research?  Do you think there are any negative consequences to adhering to these principles? |
| **Skills** | What skills do you possess that you think would be helpful in adhering to these GCP principles?  What skills do you want to possess or improve upon in these areas? |
| **Environmental context and resources** | Is there anything about your current context or environment that makes adhering to these principles easier or more difficult? Please give examples.  Do you have the necessary resources for those expected to undertake research in this area? (Prompt: education, training, information, time off work, people) |
| **Beliefs about capabilities** | How confident are you about being able to adhere to the GCP principles when conducting research? |
| **Memory, attention, decision processes** | What things might affect your decision processes or ability to remember to adhere to GCP principles? |
| **Optimism** | How confident are you that you will be able to increase your skills/ability to adhere to the principles of GCP in research? |
| **Support needs** | What ideas, if any, can you suggest regarding how the health service can support clinicians to adhere to GCP within allied health? |
|  | What additional resources do you think would support clinicians to adhere to principles of GCP when conducting research would be helpful? |
|  | What types of resources or format would be most helpful and why? (e.g., online, face to face) |
|  | What do you think is needed to make these suggestions sustainable? |
