# Supplementary material for: “How do I keep this live in my mind?” Allied Health Professionals’ perspectives of barriers and enablers to implementing good clinical practice principles in research: a qualitative exploration
Source: BMC Health Serv Res. 2023 Mar 30;23:309. doi: 10.1186/s12913-023-09238-5 (PMC10064695; doi:10.1186/s12913-023-09238-5)
Supplement: Supplementary file 3 — Supplementary Material 3 [file 12913_2023_9238_MOESM3_ESM.docx]

| **Supplementary File 3: Subcategories of reported barriers and enablers to GCP adherance according to TDF** |  |  |  |
| --- | --- | --- | --- |
| **TDF Domain** | **Enabler/Barrier subcategories** | Number of Sources | Total number of mentions |
| **Behavioural regulation** | Enablers |  |  |
|  | GCP and ethics help guide conduct | 2 | 2 |
| **Beliefs about Capabilities** | Enablers |  |  |
|  | Training helped confidence | 3 | 3 |
|  | Feeling confident or perception of not being difficult | 9 | 17 |
|  | Barriers | 0 | 0 |
|  | Less confident about aspects of training | 6 | 7 |
|  | knowledge in ethical processes | 1 | 1 |
|  | Don't feel highly confident | 3 | 4 |
|  |  |  |  |
| **Beliefs about Consequences** | Enablers |  |  |
|  | Perception of no negative outcomes of adhering | 4 | 4 |
|  | Mitigating safety risks | 9 | 10 |
|  | Improved rigour, replicability & ethical conduct | 9 | 12 |
|  | Can have consequences to your reputation as researcher if don't adhere | 2 | 3 |
|  | Barriers |  |  |
|  | Some duplication of work | 1 | 3 |
|  | Project low risk or design so not as applicable to GCP | 2 | 2 |
|  | People engaging in superficial way | 2 | 3 |
|  | Can restrict logistics of some research or too much red tape | 4 | 7 |
|  |  |  |  |
| **Emotions** | Enablers | 0 | 0 |
|  | Feeling of responsibility | 3 | 3 |
|  | Feel proud adhering to international standard | 2 | 3 |
|  | Feel comforted or reassured structures in place | 3 | 3 |
|  | Barriers |  |  |
|  | Feels tedious or too much red tape | 2 | 3 |
|  | Feel Pressured or overwhelmed | 4 | 5 |
|  | Fear | 5 | 13 |
|  | Worry will make a mistake | 5 | 5 |
|  | Fear of too much red tape | 1 | 1 |
|  | Fear of being audited | 1 | 5 |
|  | Fear ethics will close project or other consequences | 3 | 5 |
| **Environmental Context and Resources** | Enablers |  |  |
|  | Research culture and infrastructure |  |  |
|  | Research well governed and monitored | 1 | 1 |
|  | Established research culture | 1 | 1 |
|  | Access to training and resources from   Research Office | 3 | 3 |
|  | Protected time | 1 | 1 |
|  | Project context factors |  |  |
|  | Open & regular communication with team | 2 | 2 |
|  | Established processes around project | 1 | 1 |
|  | Differing needs based on project type | 2 | 2 |
|  | Barriers |  |  |
|  | Time or Funding |  |  |
|  | Time to conduct research according to GCP around clinical or other priorities | 9 | 16 |
|  | Time to attend training | 4 | 4 |
|  | Funding to do research | 2 | 2 |
|  | Finding backfill to give time to do research | 1 | 1 |
|  | Physical Resources |  |  |
|  | Research resources not as accessible at some sites | 1 | 2 |
|  | Physical or digital space to store data | 1 | 2 |
|  | HIRRO website not user friendly | 1 | 1 |
|  | Don't know where resources or templates are | 2 | 2 |
|  | Lack of Personnel Support | 2 | 2 |
|  | Current training not specific to own project or useful | 3 | 6 |
|  | Changing environment | 1 | 1 |
|  |  |  |  |
| **Goals** | Enablers |  |  |
|  | Wants to adhere to GCP or finds important | 9 | 11 |
|  | Want to learn more skills in GCP | 2 | 2 |
|  | Want research to be credible, conducted properly | 4 | 4 |
| **Intentions** | Enablers |  |  |
|  | Reflect on previous projects | 1 | 2 |
|  | Plans to implement GCP in future projects | 3 | 3 |
|  | Being more aware or cautious of how to conduct research | 3 | 4 |
| **Knowledge** | Enablers |  |  |
|  | Training increased knowledge of GCP, best if done early | 7 | 13 |
|  | Good knowledge of what is good research conduct or GCP | 9 | 12 |
|  | Aware of concepts from Uni courses and RHD | 3 | 5 |
|  | Barriers |  |  |
|  | Unfamiliar with concept of GCP but feel doing in research | 7 | 10 |
|  | Unclear what some of the principles mean in practical way | 5 | 7 |
|  | Novice researcher- don't know what don't know | 4 | 8 |
| **Memory, Attention and Decision processes** | Enablers |  |  |
|  | Is part and parcel of doing research | 1 | 4 |
|  | Have training to support | 3 | 4 |
|  | Have processes set up ready to go | 4 | 4 |
|  | Comes naturally or automatically over time | 4 | 6 |
|  | Being more mindful of GCP with greater visibility and reminders | 4 | 7 |
|  | Barriers |  |  |
|  | Multiple people in team all doing things slightly differently | 2 | 2 |
|  | Don't remember to do reporting | 1 | 2 |
|  | Difficulties remembering training | 3 | 3 |
|  | Difficulties remembering or keeping in forefront of mind | 3 | 5 |
| **Optimism** | Enablers |  |  |
|  | General optimism | 5 | 5 |
| **Reinforcement** | Enablers |  |  |
|  | Learning from examples of poorly done research of what not to do | 3 | 3 |
| **Skills** | Enablers |  |  |
|  | Skills in consent process | 1 | 2 |
|  | Earlier research-uni training drilled principles | 2 | 3 |
|  | Personal attributes or skills |  |  |
|  | Open about what don't know to seek help | 2 | 2 |
|  | Good learner | 1 | 2 |
|  | Detail orientated, systematic | 7 | 7 |
|  | Learn as you go along | 3 | 3 |
|  | Current skills in clinical role around risk and safety assessment and confidentiality | 4 | 7 |
|  | Barriers |  |  |
|  | Skills in recruitment and informed consent process | 4 | 6 |
|  | Skills in protocol writing | 1 | 1 |
|  | Skills in mitigating risks | 1 | 1 |
|  | Recording and analysing data | 1 | 1 |
| **Social Influences** | Enablers |  |  |
|  | Teams shares responsibility | 1 | 2 |
|  | Research Fellows | 4 | 6 |
|  | PhD Supervisors | 3 | 3 |
|  | Manager | 3 | 3 |
|  | Help from people experienced in research | 6 | 13 |
|  | Ethics or Research Office | 7 | 11 |
|  | Colleagues | 7 | 8 |
|  | Barriers |  |  |
|  | Less support knowledge and experience in professional team | 2 | 2 |
|  | Different people in team may not know or follow GCP principles | 3 | 4 |
| **Social/Professional Role and Identity** | Enablers |  |  |
|  | Responsibility of PI to oversee | 8 | 10 |
|  | Aligned with values to do the right thing | 5 | 6 |
